# Health care professionals’ perceptions of unprofessional behaviour in the clinical workplace

**DOI:** 10.1371/journal.pone.0280444

**Published:** 2023-01-19

**Authors:** Kirsten F. A. A. Dabekaussen, Renée A. Scheepers, Erik Heineman, Adam L. Haber, Kiki M. J. M. H. Lombarts, Debbie A. D. C. Jaarsma, Jo Shapiro

**Affiliations:** 1 Department of Surgery, University Medical Center Groningen, Groningen, The Netherlands; 2 Department of Surgery, Brigham and Women’s Hospital, Harvard Medical School, Boston, Massachusetts, United States of America; 3 Erasmus School of Health Policy and Management, Rotterdam, The Netherlands; 4 Department of Environmental Health, Harvard T. H. School of Public Health, Boston, Massachusetts, United States of America; 5 Professional Performance & Compassionate Care Research Group, Department of Medical Psychology, Amsterdam UMC, University of Amsterdam, Amsterdam, The Netherlands; 6 Faculty of Veterinary Medicine, Utrecht University, Utrecht, The Netherlands; 7 Harvard Medical School, Boston, Massachusetts, United States of America; Imam Abdulrahman Bin Faisal University, SAUDI ARABIA

## Abstract

**Background:**

Unprofessional behaviour undermines organizational trust and negatively affects patient safety, the clinical learning environment, and clinician well-being. Improving professionalism in healthcare organizations requires insight into the frequency, types, sources, and targets of unprofessional behaviour in order to refine organizational programs and strategies to prevent and address unprofessional behaviours.

**Objective:**

To investigate the types and frequency of perceived unprofessional behaviours among health care professionals and to identify the sources and targets of these behaviours.

**Methods:**

Data was collected from 2017–2019 based on a convenience sample survey administered to all participants at the start of a mandatory professionalism course for health care professionals including attending physicians, residents and advanced practice providers (APPs) working at one academic hospital in the United States.

**Results:**

Out of the 388 participants in this study, 63% experienced unprofessional behaviour at least once a month, including failing to respond to calls/pages/requests (44.3%), exclusion from decision-making (43.0%) and blaming behaviour (39.9%). Other monthly experienced subtypes ranged from 31.7% for dismissive behaviour to 4.6% for sexual harassment. Residents were more than twice as likely (OR 2.25, p<0.001)) the targets of unprofessional behaviour compared to attending physicians. Female respondents experienced more discriminating behaviours (OR 2.52, p<0.01). Nurses were identified as the most common source of unprofessional behaviours (28.1%), followed by residents from other departments (21%).

**Conclusions:**

Unprofessional behaviour was experienced frequently by all groups, mostly inflicted on these groups by those outside of the own discipline or department. Residents were most frequently identified to be the target and nurses the source of the behaviours. This study highlights that unprofessional behaviour is varied, both regarding types of behaviours as well as targets and sources of such behaviours. This data is instrumental in developing training and remediation initiatives attuned to specific professional roles and specific types of professionalism lapses.

## Introduction

Professionalism can be defined as behaviours that support interpersonal and organizational trust [1]. Unprofessional behaviour threatens patient safety by negatively impacting interprofessional communication, psychological safety and the clinical learning environment [2, 3], which can lead to adverse events, errors and even increased patient mortality [4–6]. In addition, unprofessional behaviour undermines the wellbeing of health care providers by increasing self-doubt, lowering morale and contributing to burnout [7, 8].

Previous literature shows that most healthcare providers behave professionally [9, 10], but the detrimental influence of those who do not far outweighs their numbers [11]. A survey of 102 US health care facilities reported 77% of health care professionals having witnessed physicians’ unprofessional behaviour and 65% witnessing this at least five to six times per year [2]. In the peri-operative setting, unprofessional behaviour was witnessed by 35–75% of health care providers [9, 12]. Accrediting organizations have been holding medical schools, hospitals and other healthcare organizations accountable for improving the level of professionalism and stopping the mistreatment of students and other learners [13–15]. In 2008, the Joint Commission mandated ending all behaviours that “undermine a culture of safety” [13]. Meeting these goals, however, remains challenging [14, 16, 17].

Alarmingly, bullying and harassment of medical students, residents and other healthcare team members remains highly prevalent worldwide [18–20]. Some studies have looked at specific types of unprofessional behaviour, such as harassment and discrimination towards medical students and residents [18, 19], bullying towards residents [20], and yelling and bullying behaviours in the peri-operative setting [12, 14]. Other studies have described the specific sources and/or targets of unprofessional behaviours [12, 21]. As unprofessional behaviour is not a monolithic problem, the identification of different manifestations of unprofessional behaviour across different professional roles and departments is necessary to adequately address the challenge [16, 22]. Our study is unique in examining multiple types of unprofessional behaviour, detailing both the sources and targets of each type of behaviour as well as differentiating whether those behaviours are more likely to occur within the same department or are rather inter-departmental.

The study (i) investigates the frequency and types of unprofessional behaviour and (ii) identifies those health care professionals most likely to exhibit unprofessional behaviours as well as those most likely to be the targets. The data can be used to develop training and remediation processes attuned to the specific type of professionalism lapses and focused on the specific challenges per professional role.

## Methods

### Setting and participation

This study was set in one academic medical center in the United States, the Brigham and Women’s Hospital (BWH), a 793-bed tertiary care facility that serves as a major teaching hospital of Harvard Medical School. From 2008–2019, BWH had a Center for Professionalism and Peer Support (CPPS), the mission of which was to establish a culture of trust and respect throughout the organization. The organization developed a novel, safe and fair process for reporting, assessing, and addressing concerns related to either patterns or egregious incidents of unprofessional behaviours [1]. Educational and training programs were designed to mitigate unprofessional behaviours. These training sessions used simulation-based video cases of various subtypes of unprofessional behaviours with actors representing various professional roles and were mandatory for all credentialed employees (attending physicians, residents and advanced practice providers (APPs)) [23]. APPs included nurse practitioners, physician assistants, clinical nurse specialists, certified registered nurse anesthetists and certified nurse midwifes. For this study, these educational and training programs were used as a convenience sample technique to distribute the survey to facilitate a high response rate as surveys were handed out and completed at the start of the course as well as guaranteeing a representative sample due to the obligatory nature of the training sessions. All APPs, residents and attending physicians working at BWH qualified to participate in the study. The decision to require this specific training only for these groups was based on resource constraints. Professionals with different professional roles were therefore not included in this study.

### Data collection

All credentialed employees were notified through an online educational system of their required participation in one of the monthly professionalism training sessions for which they electronically registered. All attending physicians, residents and APPs were required to attend one of those training sessions, and each participant signed up randomly at their convenience. Participants were not given any description of the specifics of the training program. We collected data immediately prior to the commencement of the mandatory professionalism training session to prevent information bias. All sessions in this study were led by the same trainer (JS). Participants were provided with a brief verbal description and aims of the study and invited to complete an anonymous paper survey. Participants were not able to attend the training session more than once, which prevented repeat participants in the dataset. We ensured privacy during the collection of the surveys: all surveys were anonymous; participants were not able to see responses from other attendees; nor was the session trainer able to correlate surveys with individual respondents. Time was given at the beginning of the training session to allow participants to fill out the surveys. The paper surveys were collected at the end of each session and entered in an excel digital database for further analysis. The excel dataset was then imported into the statistical software program R statistical software package 3.6.0 to perform the analysis. Participants were not incentivized to participate in the study. The Institutional Review Board of Partners Healthcare waived ethical approval for this study (2017P000702/PHS).

### Survey development

The survey was based on a previous workplace behaviour survey developed in 2011 through an extensive literature search of unprofessional behaviours reported in healthcare organizations, the examples of “behaviours that undermine a culture of safety” as detailed by The Joint Commission [13], as well as the senior author’s (JS) experiences as director of the CPPS in dealing with professionalism lapses [1]. Face validity was established by asking a panel of expert colleagues to critically review the survey and then adapting the items according to this feedback. Next, the survey was evaluated in several group discussions of trainees and attending physicians who shared their interpretation of the meaning of each item. The items were then rephrased for interpretative clarity. The resulting survey was used in practice for six years [21]. In 2017, the original survey was modified to allow for a more in-depth analysis of the frequency of various subtypes of unprofessional behaviours identified in the literature [15, 22, 24, 25] and by the senior author (JS). A new version of the survey including these behavioural subtypes was discussed in an expert panel group consisting of a patient safety expert, a safety culture expert, a medical education expert and two physicians during a conference call. Experts were asked to assess the clarity and relevance of the questions. The feedback was incorporated and a second version of the updated survey was sent to all experts electronically for final approval. The following behavioural subtypes were included: excluding from decision-making; failing to respond to calls/pages/requests; blaming; dismissing; yelling or other displays of anger; sexual harassment; and discrimination based on gender, sexual orientation, race/ethnicity, religion, age, disability, color, national origin, pregnancy, or genetic information.

For this study, we defined “experiencing unprofessional behaviours” as either witnessing or being the target of unprofessional behaviours. Participants were instructed to reflect on their own work environment. The frequency of multiple types of behaviours was indicated on a scale from daily–weekly–monthly–annually–never. The survey included six items requiring single or multiple closed answers (S1 Fig).

### Data analysis

First, we calculated the general frequency of unprofessional behaviours experienced by the respondents and the frequencies of the behavioural subtypes. Next, we used regression models to explore associations between different professional roles, gender, and types of unprofessional behaviours. We modelled the qualitative report of frequency (daily, weekly etc.) as an ordinal variable. The p-value was computed with a Wald test after an ordinal logistic regression. For gender related analyses only, we excluded from analysis the group of respondents that did not wish to answer the gender question, because of its small size. We used an ordinal regression analysis to identify the sources of unprofessional behaviours and a negative binominal regression to determine the rate at which unprofessional behaviours were exhibited by different professional roles. P-values were again computed using the Wald test. Significance for all tests was defined as P <0.05. Statistical analysis was performed using the R statistical software package 3.6.0. R markdown code to reproduce the analysis can be provided on request.

### Results

A total of 388 surveys were available for analysis. The response rate is estimated at > 95%. based on the trainer comparing the number of completed surveys to the approximate number of participants in each session. Out of the 388 respondents, 211 (54.3%) respondents self-identified as female, 165 self-identified as male (42.5%) and 12 (3.0%) did not wish to answer. The professional roles of the respondent group consisted of 118 attending physicians (30.4%), 169 residents (43.6%), and 101 advanced practice providers (26.0%) (S2 Fig).

### Frequencies of unprofessional behaviour and its subtypes

**Fig 1:**
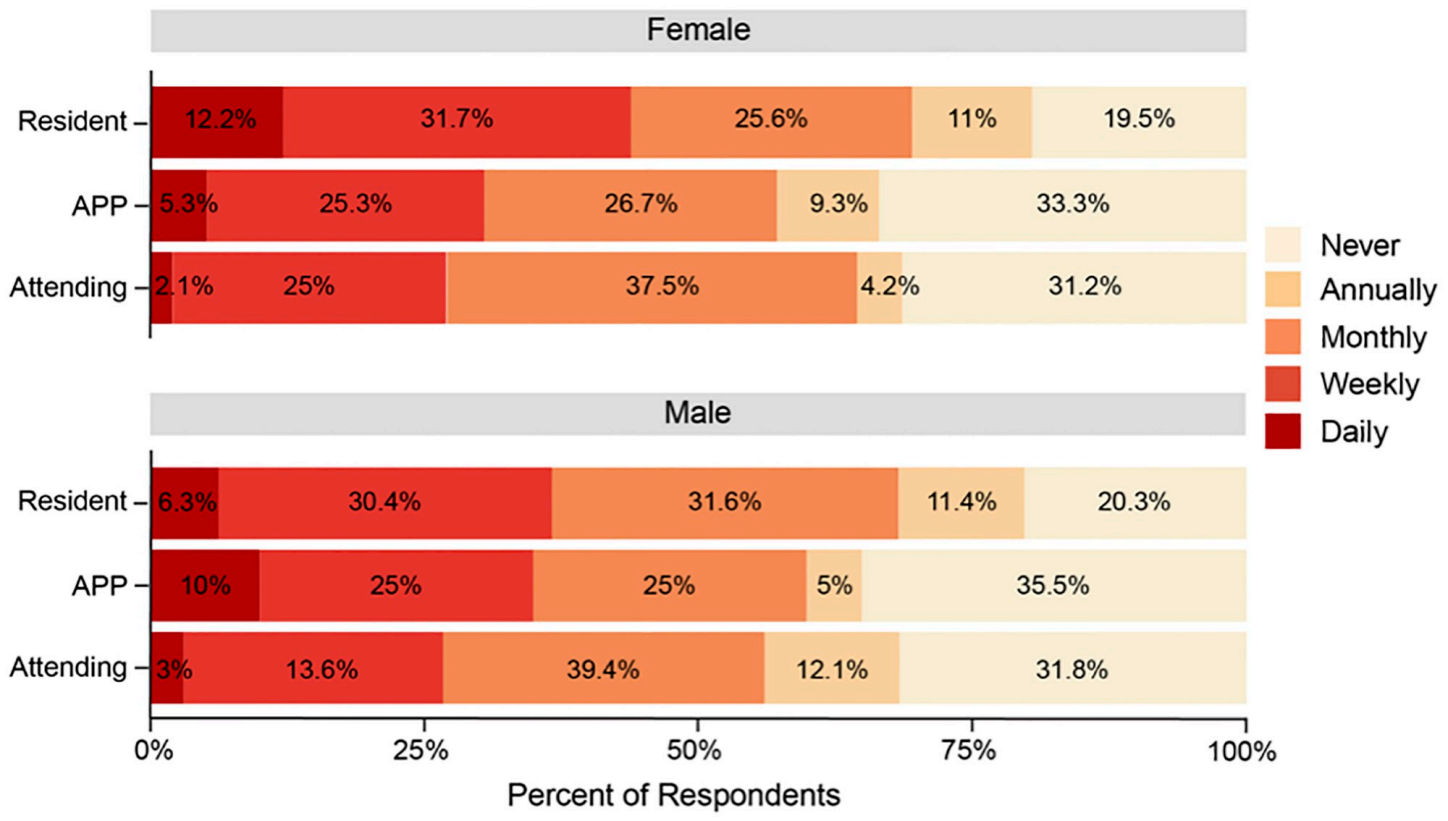
Role and gender differences in experienced frequencies of unprofessional behaviour. Frequency distribution of experienced unprofessional behaviour differentiated per professional role and gender. Data are expressed as percentage of respondents. Percentages have been rounded and may not total 100. Abbreviations: Attending, Attending physician. APP, Advanced Practice Provider.

**Fig 2:**
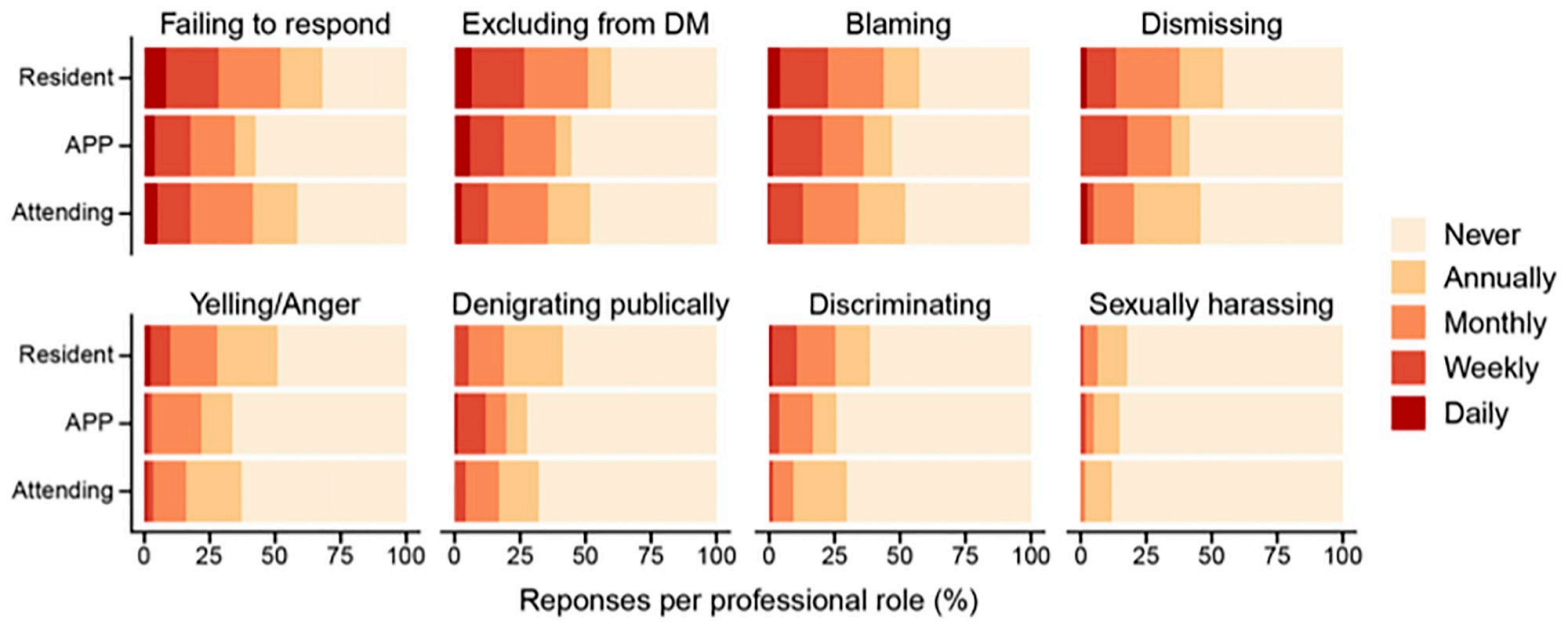
Frequencies of unprofessional behavioural subtypes experienced per professional role. Frequency distribution of different types of unprofessional behaviour experienced among different professional roles. Absolute numbers and corresponding percentages for all frequencies (never, annually, monthly, weekly, daily) are presented in the table structured by professional role. Abbreviations: Attending, Attending physician, DM, decision-making.

In general, 244 (63%) respondents experienced unprofessional behaviours at least once per month, with 26 (6.8%) experiencing it daily, 99 (25.5%) weekly and 119 (30.7%) monthly. Thirty nine (10%) of respondents experienced unprofessional behaviours annually, and 105 (27.0%) reported never experiencing unprofessional behaviours (S3 Fig). Frequency distributions of unprofessional behaviours per professional role and gender are shown in Fig 1. The frequencies of the different behavioural subtypes were analysed separately (Fig 2 and S4 Fig).

When analysing the frequencies of unprofessional behavioural subtypes experienced once a month or more, the most frequently experienced behaviour was colleagues failing to respond to pages/calls/requests, with 44.3% of respondents experiencing this at least once a month, 6.2% experiencing it daily, 16.2% weekly and 21.9% monthly. The second most frequently reported subtype was exclusion from decision-making (43.0%), with 5.1% experiencing it daily, 15.2% weekly and 22.7% monthly. From all respondents, 39.4% experienced blaming behaviour once a month or more, with 2.8% experiencing it daily, 16.7% weekly and 19.9% monthly.

Dismissive behaviour (48.5%), yelling or (other) types of anger (42.4%) and denigrating publicly (35.1%) were reported once a month or more frequent (Fig 2). At least once a month, 18.3% of respondents experienced discrimination and 4.6% experienced sexual harassment. Females were significantly more likely to experience discrimination than males (OR 2.52, CI 1.337, 4.765), p < 0.01) (S5 Fig).

### Professional role differences in experiencing specific types of unprofessional behaviour

**Fig 3:**
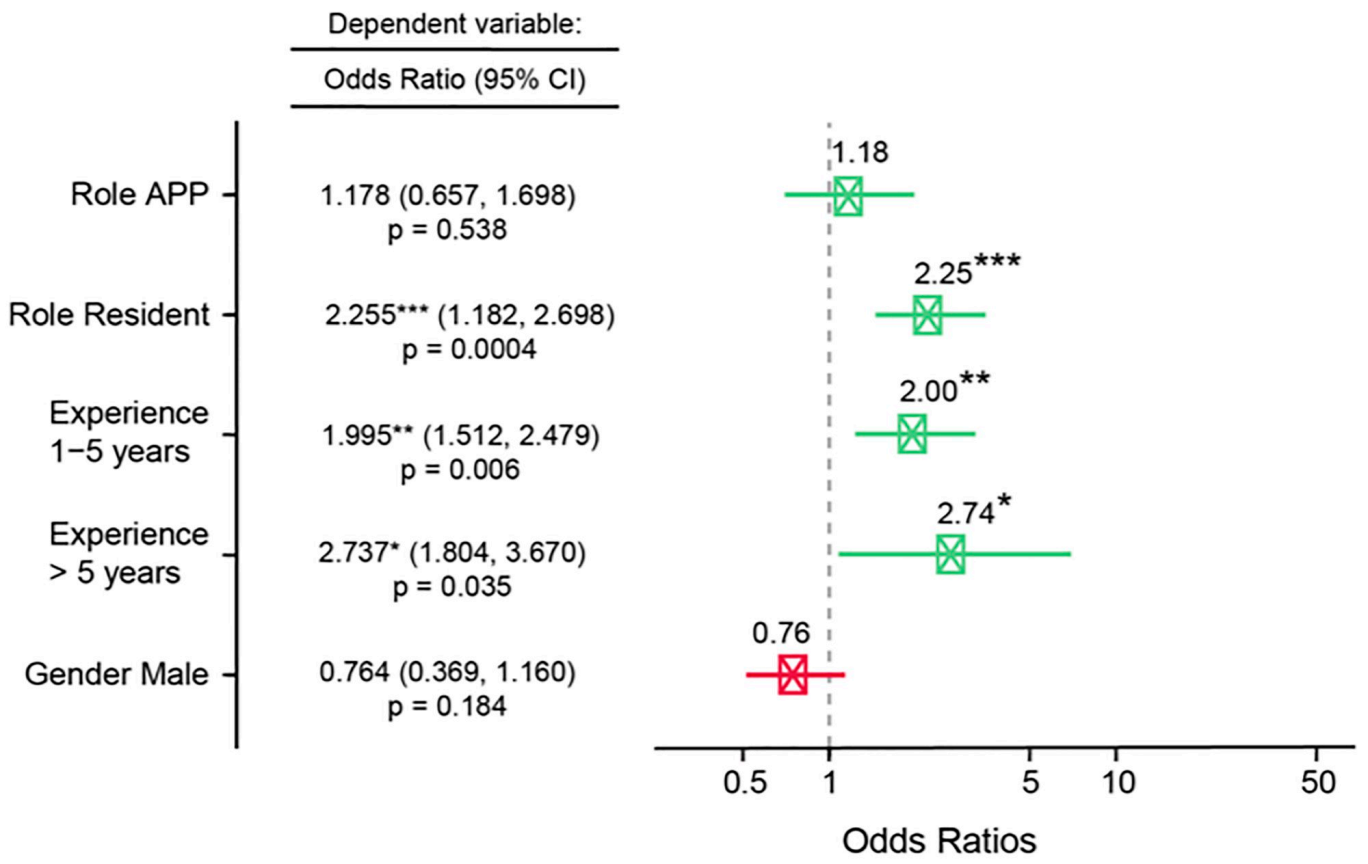
Factors influencing the likelihood of experiencing unprofessional behaviour. Ordinal logistic regression coefficients demonstrating the association of professional role, time working at the institution (experience) and gender on the likelihood of experiencing unprofessional behaviour. Reference groups selected: “attending physicians” for professional role, “<1 year” for time worked at institution and “females” for gender. Positive associations are shown in green, negative in red. Odds ratios were computed with an ordinal logistic regression. P-values were computed with a Wald test and significance was determined using * p < 0,05, ** p < 0.01, *** p < 0,001. Error bars indicate 95% confidence intervals. Abbreviations: APP, Advanced practice provider.

Residents experienced unprofessional behaviours significantly more frequently (OR 2.25, CI 1.182, 2.698, p<0.001) compared to attending physicians (Fig 3). The majority of female (69.5%) and male (68.3%) residents experienced those behaviours at least once per month (Fig 1). APPs were significantly more likely to experience dismissive behaviour (OR 2.44, CI 1.585, 3.291, p<0.05) compared to attending physicians (S6 Fig). Respondents working 1–5 years at the organization were twice (OR 2.0, CI 1.512, 2.479, p<0.1) as likely and respondents working > 5 years even more likely (OR 2.74, CI 1.804, 3.670 p<0.05) to experience unprofessional behaviours compared to respondents working < 1 year at the organization (Fig 3).

### Sources of unprofessional behaviours

**Fig 4:**
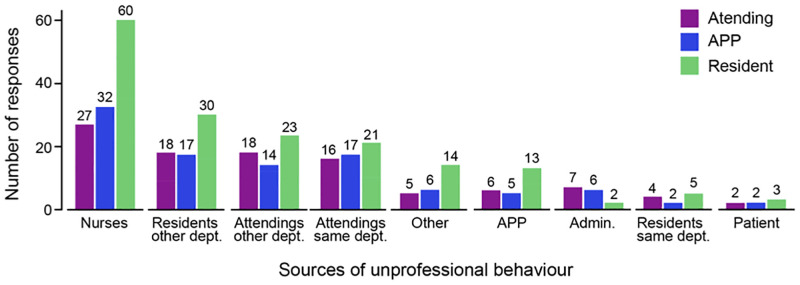
Sources versus target distribution of unprofessional behaviour. Sources that exhibit the unprofessional behaviour versus the targets that experience the unprofessional behaviours per professional role. The coloured bars represent the target groups (Attending physicians, APPs, Residents) and the groups listed in the x-axis represent the sources of unprofessional behaviour. “Other” includes technicians, medical assistants and researchers. Abbreviations: DEPT, department, Attending, Attending physician, APP, Advanced practice provider, Adm., Administrator.

Participants were then asked which professional roles were the most frequent source of unprofessional behaviours and could select more than one professional role. In total, 375 roles were identified by the participants, out of which 119 (31.7%) respondents identified nurses, 62 (16.5%) identified residents from other departments and 58 (15.5%) identified attending physicians from other departments. When assessing the interdepartmental distribution, respondents experienced the behaviours more frequently from residents/attending physicians in other departments (120, 32%)) than from residents/attending physicians within their department (65, 17,3%). The perception of the sources of unprofessional behaviours were mostly consistent amongst all the respondents’ roles (Fig 4). Compared to attending physicians, patients (IRR 0.059, CI -1.367, 1.485, p<0.001) were the least likely to be identified as source of unprofessional behaviours, followed by APPs (IRR 0.176, CI 0,691, 1.044, p<0.001) and administrators (IRR 0.206, CI -0,608, 1.019, p = 0.001). Compared to females, males were significantly less likely to be targets overall (IRR 0.61, CI 0.318, 0.903, p < 0.01) (S7 Fig).

## Discussion

Our findings show that unprofessional behaviour was experienced frequently by attending physicians, residents and APPs and was mostly inflicted on these groups by those outside their discipline or department. Residents were more than twice as likely to experience unprofessional behaviours compared to attending physicians, and nurses were most frequently identified as the source of unprofessional behaviour, followed by residents from other departments. Women were significantly more likely to experience discriminating behaviour and to be targets of unprofessional behaviour overall.

Many of our findings are related to tensions and conflicts between various professional roles, disciplines, and departments. Modern healthcare is delivered by multi-disciplinary healthcare teams who rely on effective teamwork and communication to ensure safe and high-quality patient care [26]. It is known that interdisciplinary teamwork is challenged by the often poor understanding of and respect for each other’s roles and contributions [26, 27]. Social identity theory explains that members of a professional group (e.g. physicians, nurses or allied health professionals) tend to see the attributes of their group as positive and those of other groups as less desirable [28, 29]. The finding that exhibitors of unprofessional behaviour more frequently behave unprofessionally towards health care team members from other departments, indicates a strong “ingroup” and “outgroup” distinction, suggesting that healthcare culture is exceedingly tribal [28].

Our findings may also indicate a role conflict among residents, being reflected in residents to be often a target as well as a source of unprofessional behaviour. This role conflict may result from being caught between what the attending physicians on their service want for their patients versus what others in various disciplines believe is the best approach [30]. They often lack the conflict management skills to deal effectively with those professional tensions [31]. In addition, residents’ age-demographic, vulnerability as learners, and lack of power are likely factors that place residents at risk for being targets. Negative role-modelling by their supervisors and the fact that residents are dependent on the evaluations and opinion of their performance by their attending physicians might lead to residents exhibiting unprofessional behaviours as well [32].

With regard to gender, our findings correspond with previous literature, showing that women are more likely to be the target of unprofessional behaviour overall, and significantly more likely to experience discriminating behaviour and sexual harassment [33]. This difference is already observed in medical school, but remains a challenge for female residents and attending physicians as well [14, 15]. These experiences ratify and define the male-dominated culture in medicine and healthcare [34].

Another noteworthy finding in our study is that those surveyed perceived nurses as the most common source of unprofessional behaviour. This finding is comparable with previous studies demonstrating that the majority of health care professionals reported unprofessional behaviour from nurses, and interns identified nurses as the most common source [21, 35]. In addition to the lack of interdisciplinary training, other explanations include differences in communication training for various disciplines [30, 31, 36], historical inequities, and interprofessional hierarchies [37]. Although we could not include nurses in this study, they are known to experience many forms of unprofessional behaviour as well [5, 35, 37].

### Strengths and limitations

This study contributes to the field of knowledge by clarifying which types of unprofessional behaviour are experienced by various healthcare professionals fulfilling different professional roles in the healthcare team and in what frequency, as well as illuminating both the targets and the sources of unprofessional behaviour from the point of view of the groups studied. The level of detail this study provides can be useful in developing processes to prevent and remediate unprofessional behaviours (see Implications).

A limitation of this study is that the data was gathered by convenience sampling, which may have resulted in selection bias. Nonetheless, as the data was gathered in the context of obligatory training sessions, all health care professionals suitable for the study were invited to participate. In addition, our findings on the most frequent targets and types of unprofessional behaviour are consistent with previous research using diverse health care samples [19, 20, 38, 39]. Our study is furthermore limited by having data from only one academic hospital, which questions the generalizability of the results. That said, national accrediting organizations such as The Joint Commission, the ACGME and the LCME have all recognized that most organizations are struggling to deal with recurrent professionalism lapses [13–15]. Another limitation is that data was collected using a self-reported survey that relies on the perception of respondents and not on objectively observed unprofessional behaviour. However, the anonymity of the data collection and the fact that the organization did not depend on these anonymous surveys as a reporting mechanism lends credence to the results. Moreover, in this study we intended to investigate the prevalence of specific behaviours between different groups, rather than investigate the nature of individual professionalism concerns. As a practical matter, most studies on professionalism rely on lived experience rather than direct observation. The limitation of our reporting only an estimated response rate as described above, is mitigated by the fact that the number of participants in each session was quite small and the trainer directly counted each returned survey. A further limitation is that not all healthcare groups were included in the study sample even though these groups have their own important perceptions of unprofessional behaviour [5, 35]. While beyond the scope of our study, this is an important area for future investigation. Finally, in retrospect, it would have been valuable to include race/ethnicity as demographic information of participants to investigate the prevalence of experienced unprofessional behaviour in professionals with different racial backgrounds. This is an important area of future research.

### Implications

Organizations need a fair, safe and relational process for raising professionalism concerns so that these behaviours can be assessed and addressed [1]. Responses to such concerns should then depend on whether the concerns are validated, the specific types of professionalism lapses, and which people or groups are affected. Online safety reporting systems may not lend themselves to such a discerning and relational approach [40]. In parallel, professionalism initiatives can also be informed by understanding how various disciplines interact. Proactive interdisciplinary educational approaches could be used to train people to understand and appreciate each other’s roles and viewpoints [27] and to overcome disciplinary and departmental barriers to build relational trust and enhance communication [28, 41]. Recognizing that unprofessional behaviours may exacerbate by increasing workloads and stress, potentially leading to burnout, is needed to address the existing well-being crisis amongst healthcare professionals [42, 43]. Physicians with burnout are twice as likely to deliver suboptimal care because of low professionalism and three times more likely to receive low satisfaction ratings from patients [7]. Interventions and accountability on both the behavioural and the system level are therefore needed to manifest healthy and sustainable working environments [44–47]. Ultimately, both the organization and health care professionals are responsible for the workplace culture, and the impact of solutions will be strengthened if they come from within the medical profession [48].

## Conclusions

This study highlights that unprofessional behaviour is experienced frequently by attending physicians, residents and APPs and there are significant variations in types of unprofessional behaviours as well as the sources and targets of such behaviours. Residents are the most frequent targets and women were more likely to experience unprofessional behaviour in general compared to males. Nurses were the most frequent source of the behaviours, followed by residents from other departments. Many of the behaviours reflect the need for interdisciplinary teaching and training in communication and conflict management so that health care providers can be knowledgeable and respectful of each other’s roles and viewpoints and learn how to provide and receive proper feedback on behavioural aspects. Unprofessional behaviour is not a monolithic entity and therefore requires a comprehensive and tailored approach to improving patient safety, the workplace culture, and the well-being of health care providers.

## Supporting information

S1 Fig Workplace behaviour survey. Workplace behaviour survey distribution to all participants in the study.
(DOCX)

S2 Fig Demographics of respondents. Demographics of study sample. Data in the figure are expressed as percentage of respondents. Percentages have been rounded and may not total 100. Corresponding absolute numbers and totals are shown in the table. The group “Wish not to answer” was classified as non-binary identification of gender. Abbreviations: Attending, Attending physician.
(TIF)

S3 Fig Overall frequency of experienced unprofessional behaviour. Overall reported frequencies in experiencing unprofessional behaviour. Data are expressed as percentage of respondents. Percentages have been rounded and may not total 100.
(TIF)

S4 Fig Frequencies of unprofessional behavioural subtypes experienced by gender. Frequency distribution of different types of unprofessional behaviour experienced for males and females. Absolute numbers and corresponding percentages for all frequencies (never, annually, monthly, weekly, daily) are presented in the table structured by gender. Abbreviations: DM, decision-making.
(TIF)

S5 Fig Gender differences in experiencing specific types of unprofessional behaviour. Odds ratios, their confidence intervals and p-values estimating the interaction between gender and experiencing specific subtypes of unprofessional behaviour for males compared to females. Positive associations are shown in green, negative in red. Odds ratios were computed with an ordinal logistic regression. P-values were computed with a Wald test and significance was determined using * p < 0,05, ** p < 0.01, *** p < 0,001. Error bars indicate 95% confidence intervals. Abbreviations: Attending, Attending physician. APP, Advanced practice provider. DM, decision-making.
(TIF)

S6 Fig Professional role differences in experiencing specific types of unprofessional behaviour. Odds ratios, their confidence intervals and p-values estimating the interaction between professional role and specific subtypes of unprofessional behaviour for APPs and residents compared to attending physicians. APPs are significantly more likely to experience dismissive behaviour. All interaction terms in the model are shown, positive interactions are shown in green, negative in red. The cross in the figure represents the Odds ratio, the error bars represent the 95% confidence interval. The corresponding absolute numbers are illustrated in the table. Odds ratios were computed with an ordinal logistic regression. P-values were computed with a Wald test and significance was determined using * p < 0,05, ** p < 0.01, *** p < 0,001. Error bars indicate 95% confidence intervals. Abbreviations: Attending, Attending physician. APP, Advanced practice provider. DM, decision-making.
(TIF)

S7 Fig Incidence ratios of target groups versus sources of unprofessional behaviour. Incidence rate ratios (IRRs) and associated confidence intervals and p-values estimating interaction terms between professional role of victim and that of the perpetrator of unprofessional behaviour. All interaction terms in the model are shown and positive interactions are shown in green, negative in red. IRRs were computed using negative binominal regression. P-values were computed with a Wald test and significance was determined using * p < 0.05, ** p < 0.01, **** p < 0.001. Error bars indicate 95% confidence intervals. Abbreviations: Attending, Attending physician. APP, Advanced practice provider. DM, decision-making.
(TIF)

S1 Dataset Minimal anonymised dataset. (DAT)

## References

[1] Shapiro J, Whittemore A, Tsen LC. Instituting a Culture of Professionalism: The Establishment of a Center for Professionalism and Peer Support. *Jt Comm J Qual Patient Saf*. 2014;40(4):168–AP1. doi: 10.1016/s1553-7250(14)40022-9 24864525

[2] Rosenstein AH, O’Daniel M. A Survey of the Impact of Disruptive Behaviours and Communication Defects on Patient Safety. *Jt Comm J Qual Patient Saf*. 2008;34(8):464–471. doi: 10.1016/s1553-7250(08)34058-6 18714748

[3] Wiegmann DA, ElBardissi AW, Dearani JA, Daly RC, Sundt TM. Disruptions in surgical flow and their relationship to surgical errors: An exploratory investigation. *Surgery*. 2007;142(5):658–665. doi: 10.1016/j.surg.2007.07.034 17981185

[4] Cooper WO, Spain DA, Guillamondegui O, et al. Association of Coworker Reports About Unprofessional Behaviour by Surgeons with Surgical Complications in Their Patients. *Jama Surg*. 2019;154(9):828. doi: 10.1001/jamasurg.2019.1738 31215973 PMC6585020

[5] Saxton R, Hines T, Enriquez M. The Negative Impact of Nurse-Physician Disruptive Behaviour on Patient Safety: A Review of the Literature. *J Patient Saf*. 2009;5(3):180–183. doi: 10.1097/pts.0b013e3181b4c5d7 19927052

[6] Cochran A, Elder WB. Effects of disruptive surgeon behaviour in the operating room. Am J Surg. 2015;209(1):65–70. doi: 10.1016/j.amjsurg.2014.09.017 25454961

[7] Hodkinson A, Zhou A, Johnson J, Geraghty K, Riley R, Zhou A, et al. Associations of physician burnout with career engagement and quality of patient care: systematic review and meta-analysis. Bmj. 2022;378:e070442. doi: 10.1136/bmj-2022-070442 36104064 PMC9472104

[8] West CP, Dyrbye LN, Shanafelt TD. Physician burnout: contributors, consequences and solutions. *J Intern Med*. 2018;283(6):516–529. doi: 10.1111/joim.12752 29505159

[9] Villafranca A, Hamlin C, Enns S, Jacobsohn E. Disruptive behaviour in the perioperative setting: a contemporary review. *Can J Anaesth J Can D’anesthesie*. 2016;64(2):128–140. doi: 10.1007/s12630-016-0784-x 27900669 PMC5222921

[10] Weenink JW, Westert GP, Schoonhoven L, Wollersheim H, Kool RB. Am I my brother’s keeper? A survey of 10 healthcare professions in the Netherlands about experiences with impaired and incompetent colleagues. *Bmj Qual Saf*. 2014;24(1):56–64. doi: 10.1136/bmjqs-2014-003068 25380669

[11] Hickson GB, Pichert JW, Webb LE, Gabbe SG. A Complementary Approach to Promoting Professionalism: Identifying, Measuring, and Addressing Unprofessional Behaviours. *Acad Med*. 2007;82(11):1040–1048. doi: 10.1097/acm.0b013e31815761ee 17971689

[12] Rosenstein AH, O’Daniel M. Impact and Implications of Disruptive Behaviour in the Perioperative Arena. *J Am Coll Surgeons*. 2006;203(1):96–105. doi: 10.1016/j.jamcollsurg.2006.03.027 16798492

[13] Behaviours that undermine a culture of safety. *Sentinel Event Alert Jt Comm Accreditation Healthc Organizations*. 2008;(40):1–3. 18686330

[14] Pei KY, Hafler J, Alseidi A, Slade MD, Klingensmith M, Cochran A. National Assessment of Workplace Bullying Among Academic Surgeons in the US. *Jama Surg*. 2020;155(6). doi: 10.1001/jamasurg.2020.0263 32236505 PMC7113829

[15] Fairchild AL, Holyfield LJ, Byington CL. National Academies of Sciences, Engineering, and Medicine Report on Sexual Harassment: Making the Case for Fundamental Institutional Change. *Jama*. 2018;320(9):873–874. doi: 10.1001/jama.2018.10840 30128569

[16] Weenink J-W, Kool RB, Bartels RH, Westert GP. Getting back on track: a systematic review of the outcomes of remediation and rehabilitation programmes for healthcare professionals with performance concerns. *Bmj Qual Saf*. 2017;26(12):1004–1014. doi: 10.1136/bmjqs-2017-006710 28794242

[17] Rosenstein AH. Physician disruptive behaviours: Five-year progress report. *World J Clin Cases*. 2015;3(11):930–934. doi: 10.12998/wjcc.v3.i11.930 26601095 PMC4644894

[18] Freedman-Weiss MR, Chiu AS, Heller DR, et al. Understanding the Barriers to Reporting Sexual Harassment in Surgical Training. *Ann Surg*. 2019;Publish Ahead of Print(NA;):NA; doi: 10.1097/sla.0000000000003295 30946072

[19] Hill KA, Samuels EA, Gross CP, et al. Assessment of the Prevalence of Medical Student Mistreatment by Sex, Race/Ethnicity, and Sexual Orientation. *Jama Intern Med*. 2020;180(5):653–665. doi: 10.1001/jamainternmed.2020.0030 32091540 PMC7042809

[20] Leisy HB, Ahmad M. Altering workplace attitudes for resident education (A.W.A.R.E.): discovering solutions for medical resident bullying through literature review. *Bmc Med Educ*. 2016;16(1):127. doi: 10.1186/s12909-016-0639-8 27117063 PMC4847214

[21] Mullan CP, Shapiro J, McMahon GT. Interns’ experiences of disruptive behaviour in an academic medical center. *J Graduate Medical Educ*. 2013;5(1):25–30. doi: 10.4300/jgme-d-12-00025.1 24404222 PMC3613313

[22] Swiggart WH, Dewey CM, Hickson GB, Finlayson RAJ, Spickard WA. A Plan for Identification, Treatment, and Remediation of Disruptive Behaviours in Physicians. *Frontiers Heal Serv Management*. 2009;25(4):3–11. doi: 10.1097/01974520-200904000-00002 19603686

[23] Shapiro J; Employment Learning Innovations (ELI). Professionalism for Clinicians and Scientists. Atlanta: ELI, 2011.

[24] Fnais N, Soobiah C, Chen MH, et al. Harassment and Discrimination in Medical Training: A Systematic Review and Meta-Analysis. *Acad Med*. 2014;89(5):817–827. doi: 10.1097/ACM.0000000000000200 24667512

[25] der Vossen MM, van Mook W, van der Burgt S, et al. Descriptors for unprofessional behaviours of medical students: a systematic review and categorisation. *Bmc Med Educ*. 2017;17(1):164. doi: 10.1186/s12909-017-0997-x 28915870 PMC5603020

[26] Brock D, Abu-Rish E, Chiu C-R, et al. Interprofessional education in team communication: working together to improve patient safety. *Bmj Qual Saf*. 2013;22(5):414–423. doi: 10.1136/bmjqs-2012-000952 23293118

[27] Gittell JH, Godfrey M, Thistlethwaite J. Interprofessional collaborative practice and relational coordination: Improving healthcare through relationships. *J Interprofessional*. 2012;27(3):210–213. doi: 10.3109/13561820.2012.730564 23082769

[28] Weller J, Boyd M, Cumin D. Teams, tribes and patient safety: overcoming barriers to effective teamwork in healthcare. Postgrad Med J. 2014;90(1061):149–154. doi: 10.1136/postgradmedj-2012-131168 24398594

[29] Bochatay N, Bajwa NM, Blondon KS, et al. Exploring group boundaries and conflicts: a social identity theory perspective. Med Educ 2019;53:799–807. doi: 10.1111/medu.13881 30989682

[30] Muller-Juge V, Cullati S, Blondon KS, et al. Interprofessional collaboration on an internal medicine ward: role perceptions and expectations among nurses and residents. *Plos One*. 2013;8(2):e57570. doi: 10.1371/journal.pone.0057570 23469027 PMC3585159

[31] Foronda C, MacWilliams B, McArthur E. Interprofessional communication in healthcare: An integrative review. *Nurse Educ Pract*. 2016;19:36–40. doi: 10.1016/j.nepr.2016.04.005 27428690

[32] Sternszus R, Macdonald ME, Steinert Y. Resident Role Modeling. *Acad Med* 2016;91:427–32. doi: 10.1097/acm.0000000000000996 26579795

[33] Schlick CJR, Ellis RJ, Etkin CD, et al. Experiences of Gender Discrimination and Sexual Harassment Among Residents in General Surgery Programs Across the US. *JAMA Surg*. 2021;156(10):942–952. doi: 10.1001/jamasurg.2021.3195 34319377 PMC8319819

[34] Lombarts KMJ, Verghese A. Medicine Is Not Gender-Neutral–She Is Male. New Engl J Med. 2022;386: 1284–1287. doi: 10.1056/NEJMms2116556 35353969

[35] Rosenstein AH, Naylor B. Incidence and impact of physician and nurse disruptive behaviours in the emergency department. *J Emerg Medicine*. 2011;43(1):139–148. doi: 10.1016/j.jemermed.2011.01.019 21421291

[36] Rice K, Zwarenstein M, Conn LG, Kenaszchuk C, Russell A, Reeves S. An intervention to improve interprofessional collaboration and communications: A comparative qualitative study. *J Interprofessional*. 2010;24(4):350–361. doi: 10.3109/13561820903550713 20540614

[37] Blackwood K, Bentley T, Catley B, Edwards M. Managing workplace bullying experiences in nursing: the impact of the work environment. *Public Money Manage*. 2017;37(5):349–356. doi: 10.1080/09540962.2017.1328205

[38] Hu YY, Ellis RJ, Hewitt DB, Yang AD, Cheung EO, Moskowitz JT, et al. Discrimination, abuse, harassment, and burnout in surgical residency training. New England Journal of Medicine. 2019 Oct 31;381(18):1741–52. doi: 10.1056/NEJMsa1903759 31657887 PMC6907686

[39] Villafranca A., Hiebert B., Hamlin C. et al. Prevalence and predictors of exposure to disruptive behaviour in the operating room. Can J Anesth/J Can Anesth 66, 781–794 (2019). doi: 10.1007/s12630-019-01333-8 31168769

[40] Myers JS, Shapiro J, Rosen IM. Gotcha! Using Patient Safety Event Reports to Report People Rather Than Problems. *J Graduate Medical Educ* 2020:12:525–8. doi: 10.4300/JGME-D-20-00165.1 33149815 PMC7594777

[41] Martinez W, Lehmann LS, Thomas EJ, et al. Speaking up about traditional and professionalism-related patient safety threats: a national survey of interns and residents. *Bmj Qual Saf*. 2017;26(11):869–880. doi: 10.1136/bmjqs-2016-006284 28442609

[42] Wallace JE, Lemaire JB, Ghali WA. Physician wellness: a missing quality indicator. *Lancet Lond Engl*. 2009;374(9702):1714–1721. doi: 10.1016/S0140-6736(09)61424-0 19914516

[43] Dewa CS, Loong D, Bonato S, Trojanowski L. The relationship between physician burnout and quality of healthcare in terms of safety and acceptability: a systematic review. *Bmj Open*. 2017;7(6):e015141. doi: 10.1136/bmjopen-2016-015141 28637730 PMC5734243

[44] Panagioti M, Panagopoulou E, Bower P, et al. Controlled Interventions to Reduce Burnout in Physicians: A Systematic Review and Meta-analysis. *Jama Intern Med*. 2017;177(2):195. doi: 10.1001/jamainternmed.2016.7674 27918798

[45] Webb LE, Dmochowski RR, Moore IN, et al. Using Coworker Observations to Promote Accountability for Disrespectful and Unsafe Behaviours by Physicians and Advanced Practice Professionals. *Jt Comm J Qual Patient Saf*. 2016;42(4):149–AP3. doi: 10.1016/s1553-7250(16)42019-2 27025575

[46] Hopkins J, Hedlin H, Weinacker A, Desai M. Patterns of Disrespectful Physician Behaviour at an Academic Medical Center: Implications for Training, Prevention, and Remediation. *Acad Medicine J Assoc Am Medical Coll*. 2018;93(11):1679–1685. doi: 10.1097/acm.0000000000002126 29319539

[47] Katz MG, Rockne WY, Braga R, McKellar S, Cochran A. An improved patient safety reporting system increases reports of disruptive behaviour in the perioperative setting. *Am J Surg*. 2019;219(1):21–26. doi: 10.1016/j.amjsurg.2019.05.012 31151660

[48] Jo Shapiro. Confronting unprofessional behaviour in medicine. *BMJ* 2018;360:k1025. doi: 10.1136/bmj.k1025 29514793

