# Supplementary material for: Health care professionals’ perceptions of unprofessional behaviour in the clinical workplace
Source: PLoS One. 2023 Jan 19;18(1):e0280444. doi: 10.1371/journal.pone.0280444 (PMC9851503; doi:10.1371/journal.pone.0280444)
Supplement: S1 Fig — Workplace behaviour survey distribution to all participants in the study. (DOCX) [file pone.0280444.s001.docx]

**S1. Workplace behavior survey**

- We are asking providers, researchers and staff at BWH to complete this survey
- This survey is part of a research project focused on quality improvement within our institution
- Your participation in this survey is voluntary and all responses are anonymous
- The Principle Investigator of this research study is Jo Shapiro, MD ([jshapiro@bwh.harvard.edu](mailto:jshapiro@bwh.harvard.edu)); please feel free to contact Dr. Shapiro with any questions

**Please complete this survey based on your experience at BWH only.**

1. My main professional role is:

- Attending
- Resident or Intern (circle one)
- Allied health practitioner (NP,PA,CRNA,etc)
- Nurse
- Social worker
- Medical student
- Research PI
- Research post doc or fellow (circle one)
- Research coordinator/data manager /Research assistant
- Technician (OR, lab, other): _______________________
- Administrator/manager
- Administrative support staff
- Other (please specify): ______________

2. I have worked at BWH:

- < 1 year
- 1-5 years
- > 5 years

3. I am (circle one) MALE / FEMALE / OTHER / wish not to answer

4. In my work environment, I encounter disrespectful behavior:

- Daily
- Weekly
- Monthly
- Annually
- Never [skip to question 6]

5. Please rate how frequently you encounter the following categories of disrespectful behavior towards yourself or others:

|  | Daily | Weekly | Monthly | Annually | Never |
| --- | --- | --- | --- | --- | --- |
| Excluding from decision making |  |  |  |  |  |
| Blaming |  |  |  |  |  |
| Denigrating publically |  |  |  |  |  |
| Dismissing (e.g., hanging up the phone/ending a conversation abruptly) |  |  |  |  |  |
| Yelling or other displays of anger |  |  |  |  |  |
| Failing to respond to phone calls, pages, and/or requests |  |  |  |  |  |
| Sexually harassing |  |  |  |  |  |
| Discriminating based on gender, sexual orientation, race, religion, age, disability, color, national origin, pregnancy, or genetic information |  |  |  |  |  |

6. Choose one box in each row below:

|  | Disagree Strongly | Disagree Slightly | Neutral | Agree Slightly | Agree Strongly |
| --- | --- | --- | --- | --- | --- |
| I know where to report concerns at BWH regarding disrespectful **physician** behavior |  |  |  |  |  |
| I know where to report concerns at BWH regarding disrespectful behavior on the part of **other team members** |  |  |  |  |  |
| I am confident that BWH would address reports about disrespectful **physician** behavior |  |  |  |  |  |
| I am confident that BWH would address reports about disrespectful behavior on the part of **other team members** |  |  |  |  |  |
| BWH is actively working to enhance a culture of professionalism and respect |  |  |  |  |  |

7. The professional group that causes most of the disrespectful behavior in my work environment is:

- Attendings from my department
- Attendings from other dept
- Residents from my department
- Residents from other dept
- Allied health practitioners (NP, PA, CRNA,etc)
- Nurses
- Social workers
- Research PI
- Research post doc or fellow
- Research coordinator/data manager /Research assistant
- Technician (OR, lab, other)___________________________
- Administrators/managers
- Administrative support staff
- Other: please define: _______________
- Attendings from my department

8. Please describe two aspects of professionalism at BWH that you appreciate/value. Please note any departments, areas (units/floors/clinics), or processes that are particularly respectful or helpful in promoting professionalism.

9. Please describe two aspects of professionalism at BWH that you would like to see changed. Please note any departments, areas (units/floors/clinics), or processes that you feel are particularly problematic.

10. Other comments:
