# Supplementary figures and images for: Health care professionals’ perceptions of unprofessional behaviour in the clinical workplace
Source: PLoS One. 2023 Jan 19;18(1):e0280444. doi: 10.1371/journal.pone.0280444 (PMC9851503; doi:10.1371/journal.pone.0280444)

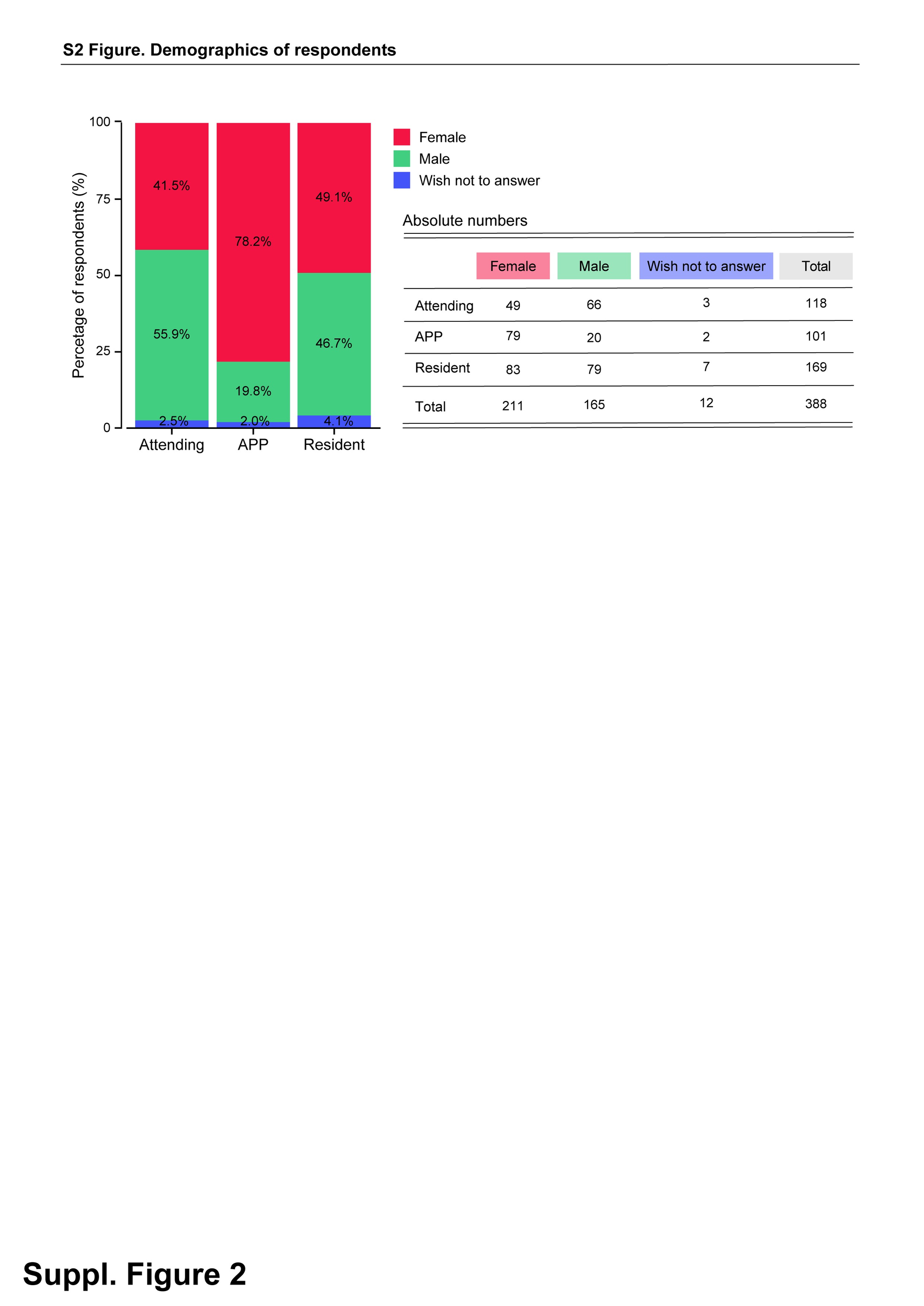

Supplement: S2 Fig — Demographics of study sample. Data in the figure are expressed as percentage of respondents. Percentages have been rounded and may not total 100. Corresponding absolute numbers and totals are shown in the table. The group “Wish not to answer” was classified as non-binary identification of gender. Abbreviations: Attending, Attending physician. (TIF) [file pone.0280444.s002.tif]

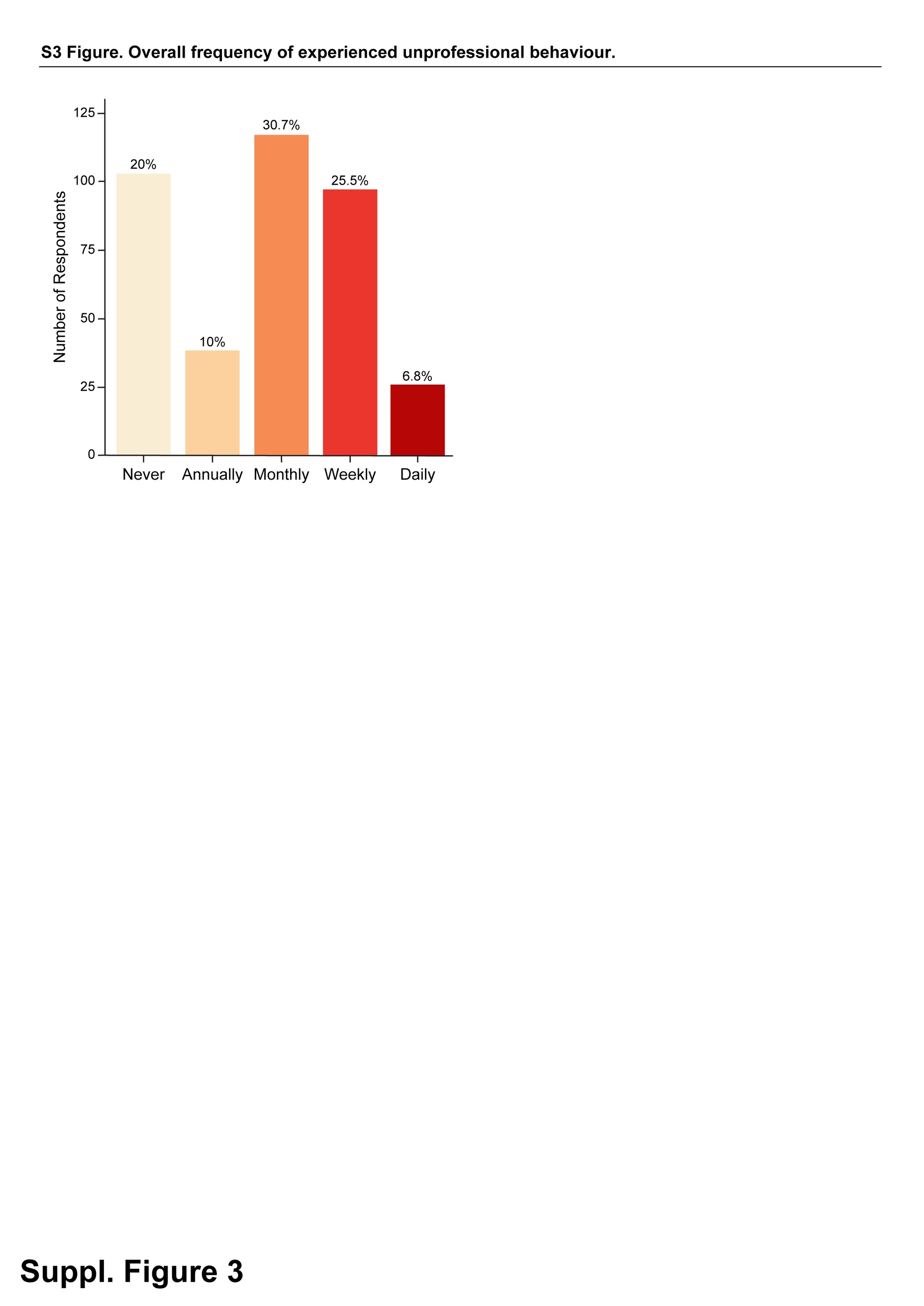

Supplement: S3 Fig — Overall reported frequencies in experiencing unprofessional behaviour. Data are expressed as percentage of respondents. Percentages have been rounded and may not total 100. (TIF) [file pone.0280444.s003.tif]

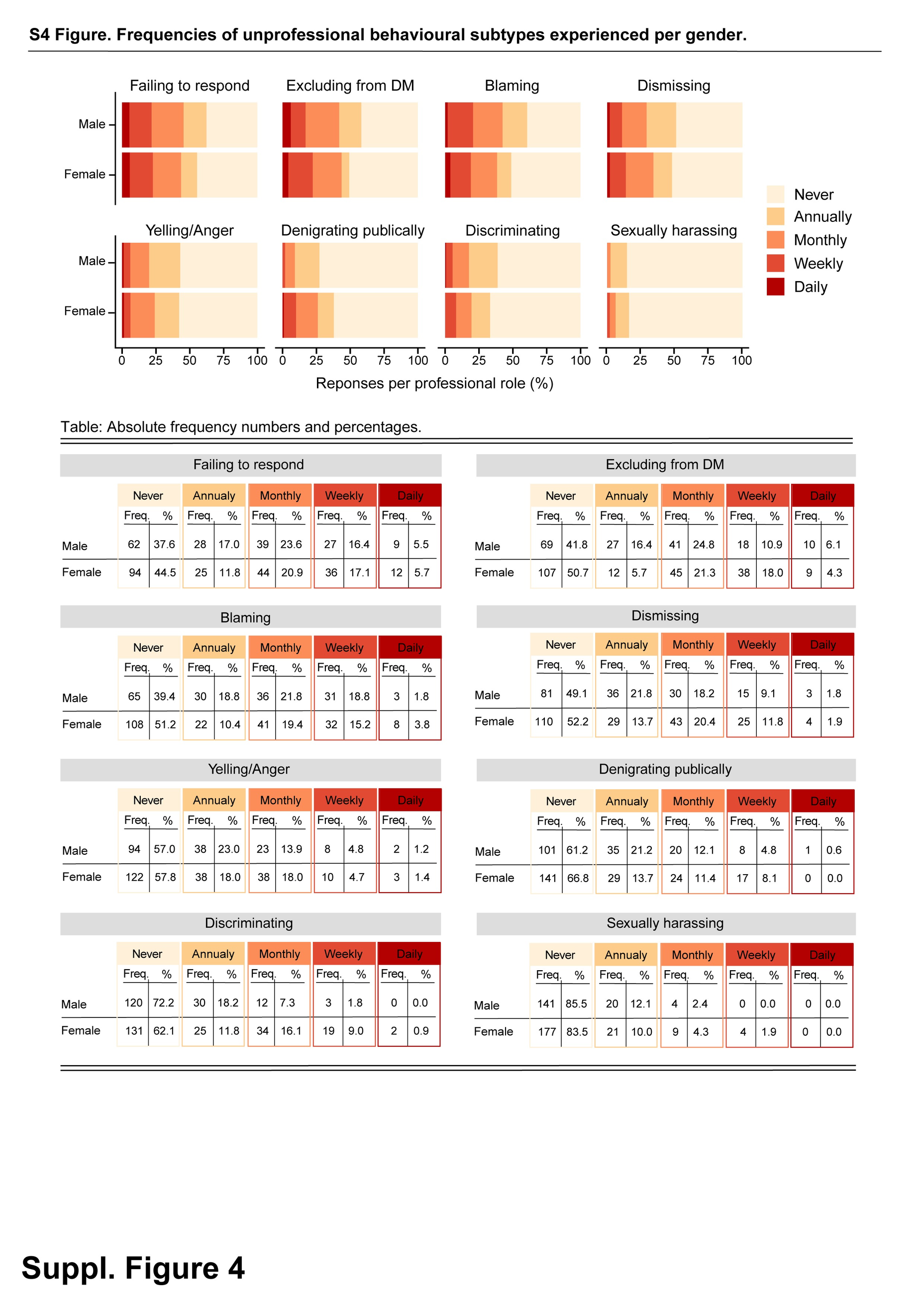

Supplement: S4 Fig — Frequency distribution of different types of unprofessional behaviour experienced for males and females. Absolute numbers and corresponding percentages for all frequencies (never, annually, monthly, weekly, daily) are presented in the table structured by gender. Abbreviations: DM, decision-making. (TIF) [file pone.0280444.s004.tif]

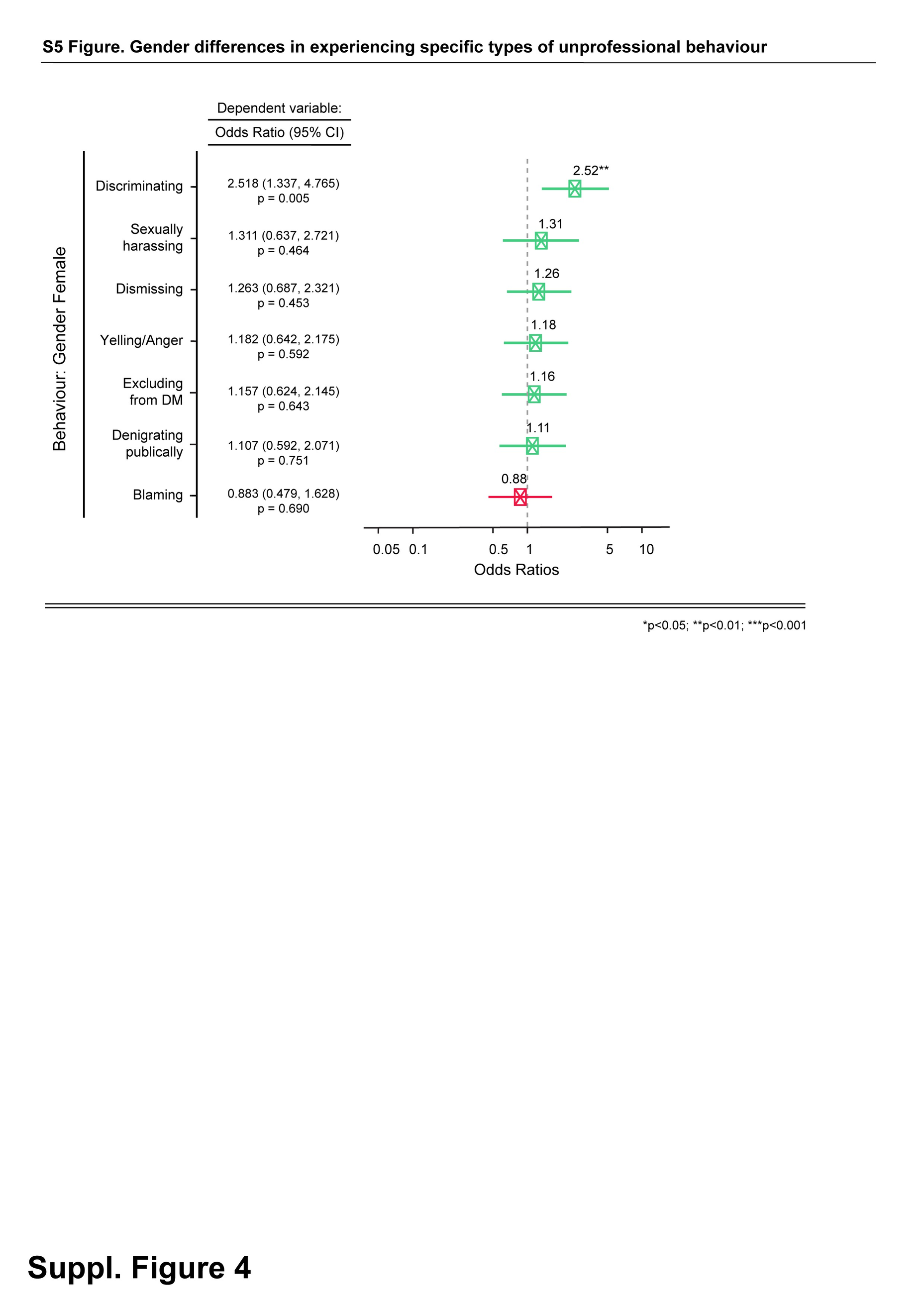

Supplement: S5 Fig — Odds ratios, their confidence intervals and p-values estimating the interaction between gender and experiencing specific subtypes of unprofessional behaviour for males compared to females. Positive associations are shown in green, negative in red. Odds ratios were computed with an ordinal logistic regression. P-values were computed with a Wald test and significance was determined using * p < 0,05, ** p < 0.01, *** p < 0,001. Error bars indicate 95% confidence intervals. Abbreviations: Attending, Attending physician. APP, Advanced practice provider. DM, decision-making. (TIF) [file pone.0280444.s005.tif]

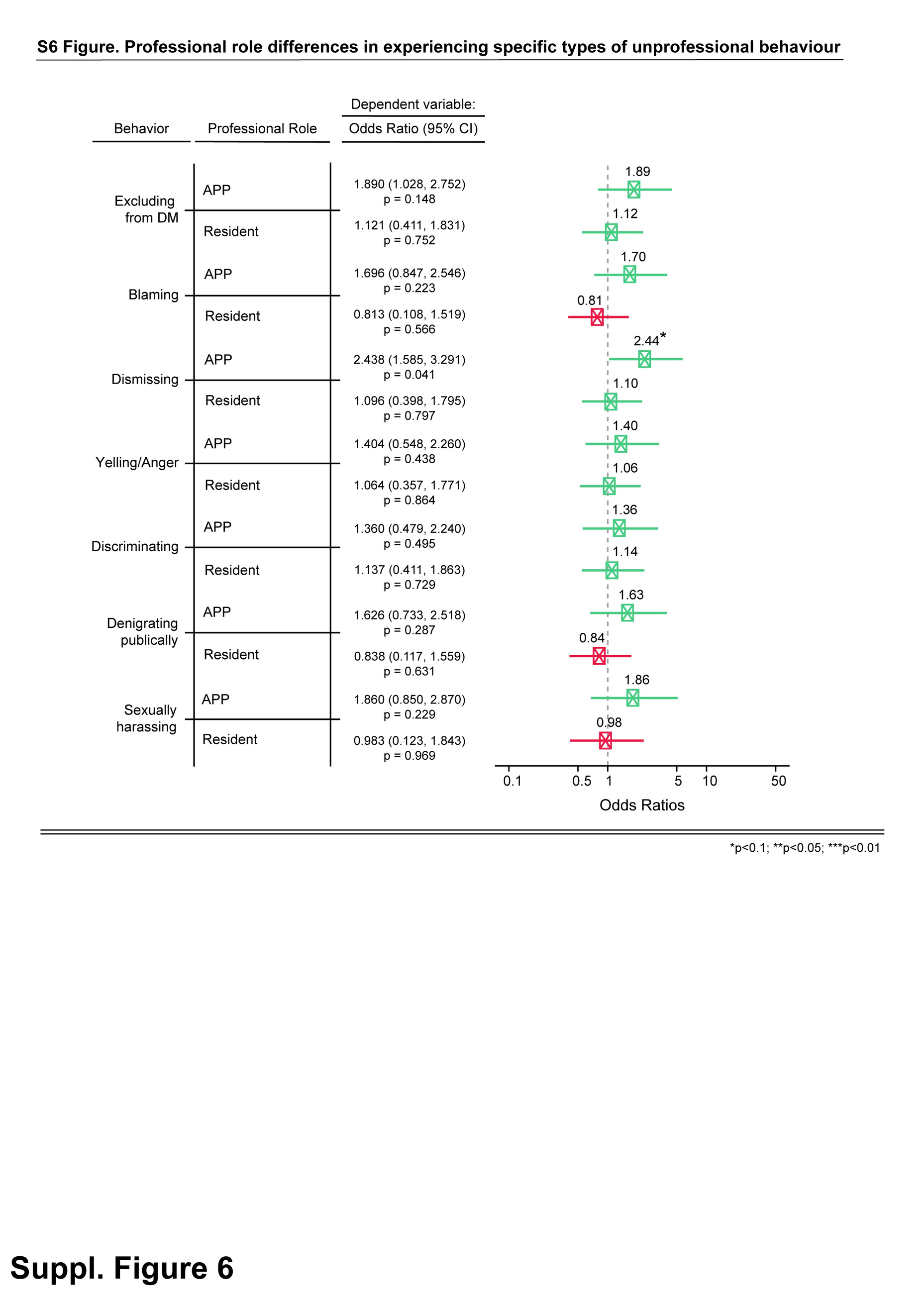

Supplement: S6 Fig — Odds ratios, their confidence intervals and p-values estimating the interaction between professional role and specific subtypes of unprofessional behaviour for APPs and residents compared to attending physicians. APPs are significantly more likely to experience dismissive behaviour. All interaction terms in the model are shown, positive interactions are shown in green, negative in red. The cross in the figure represents the Odds ratio, the error bars represent the 95% confidence interval. The corresponding absolute numbers are illustrated in the table. Odds ratios were computed with an ordinal logistic regression. P-values were computed with a Wald test and significance was determined using * p < 0,05, ** p < 0.01, *** p < 0,001. Error bars indicate 95% confidence intervals. Abbreviations: Attending, Attending physician. APP, Advanced practice provider. DM, decision-making. (TIF) [file pone.0280444.s006.tif]

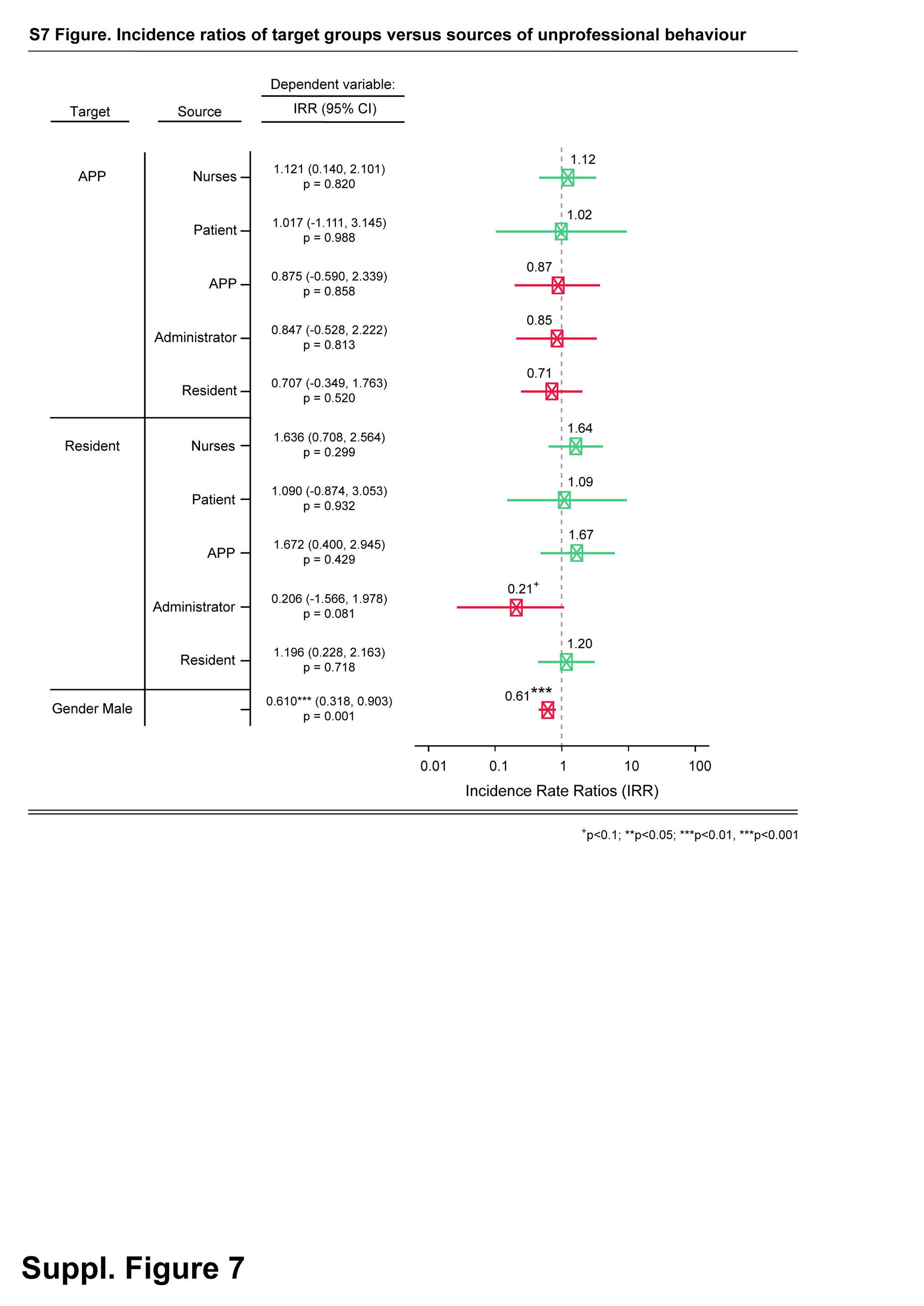

Supplement: S7 Fig — Incidence rate ratios (IRRs) and associated confidence intervals and p-values estimating interaction terms between professional role of victim and that of the perpetrator of unprofessional behaviour. All interaction terms in the model are shown and positive interactions are shown in green, negative in red. IRRs were computed using negative binominal regression. P-values were computed with a Wald test and significance was determined using * p < 0.05, ** p < 0.01, **** p < 0.001. Error bars indicate 95% confidence intervals. Abbreviations: Attending, Attending physician. APP, Advanced practice provider. DM, decision-making. (TIF) [file pone.0280444.s007.tif]
